# Diagnosis and treatment of lumbosacral discospondylitis in a calf

**DOI:** 10.1186/1746-6148-7-53

**Published:** 2011-09-13

**Authors:** Evelyne Muggli, Tanja Schmid, Regine Hagen, Barbara Schmid, Karl Nuss

**Affiliations:** 1 Department of Farm Animals, Vetsuisse-Faculty, University of Zurich, Winterthurerstrasse 260, 8057 Zurich, Switzerland; 2 Department of Small Animals, Section of Diagnostic Imaging, Vetsuisse-Faculty, University of Zurich, Winterthurerstrasse 260, 8057 Zurich, Switzerland

## Abstract

**Background:**

The aim of this case report was to describe the clinical findings, treatment and outcome of lumbosacral discospondylitis in a calf.

**Case Presentation:**

A 5.5-month-old calf was presented with difficulty in rising, a stiff and slightly ataxic gait in the hind limbs and a shortened stride. The lumbosacral region was severely painful on palpation.

Radiographic examination confirmed lumbosacral discospondylitis. Medical treatment with stall rest was instituted over six weeks. Radiographic and ultrasonographic follow-up examinations showed lysis of the endplates initially, then collapse of the intervertebral space at the lumbosacral junction and progressive sclerosis in the periphery of the lytic zones. Four weeks after institution of treatment, the calf could rise normally and the general condition gradually had returned to normal. The calf was discharged after 6 weeks and was sound at 3.5 months clinical and radiographic follow up examination. Thereafter, it was kept on alpine pastures without problems and was pregnant 1 year after the last examination.

**Conclusions:**

This report shows that recovery from lumbosacral discospondylitis is possible in heifers, provided that treatment is started before major neurologic deficits have developed and is continued for an extended period of time.

## Background

Discospondylitis is defined as inflammation and infection of an intervertebral disk accompanied by osteomyelitis of the neighbouring vertebral bodies [1,2]. This disease has been reported in many domestic animals [3-7], but is generally rare. It is diagnosed more often in dogs and pigs than in cattle and horses [8,9].

The most common cause of discospondylitis is haematogenous spread [1,10] of bacterial infection and less commonly fungal infection [1]. The disease is characterised by a chronic progressive course with clinical signs that include increased sensitivity in the area of the affected vertebrae, difficulty in rising and progressive neurological deficits, although signs may vary depending on the localisation and severity of the lesion. Other less specific signs may include pyrexia, anorexia and depression [1].

Imaging techniques such as radiography and ultrasonography are used to confirm the diagnosis [2,3,8,11].

The purpose of this case report was to describe the clinical signs, diagnostic approach, treatment and outcome of discospondylitis in a calf.

## Case presentation

A 5.5-month-old female Brown Swiss calf was referred to our clinic because of a four-week history of prolonged periods of recumbency, difficulty in rising and a stiff gait in the hind limbs. There was progressive deterioration in the calf's condition during the first two weeks of the disease followed by stationary clinical signs up to the time of referral. The appetite was normal despite intermittent fever of up to 40°C. The calf had been treated with ketoprofen (Dolovet^®^, Dr. E. Graeub AG, Switzerland) for several days on two occasions.

### a) Clinical and laboratory examination and diagnosis

At the time of admission to the large animal clinic, the calf was very quiet and its appetite was moderately reduced, but it presented with a normal mental status. The calf spent the majority of time lying in sternal recumbency when placed in a crate. When assisted, the calf stood with its hind limbs drawn forward under the abdomen, resulting in kyphosis of the back. It was reluctant to move and had a stiff and slightly ataxic gait with a shortened stride of the hind limbs. The musculature of the back and hind limbs was moderately atrophied.

Physical examination revealed abnormalities restricted to the musculoskeletal system. Palpation of the front and hind limbs, the pelvic region and tail, and cervical and thoracic vertebrae produced no abnormal findings, whereas palpation of the lumbosacral region elicited severe pain. Skin sensitivity of the entire body was normal. On transrectal examination, slight soft tissue swelling was palpated at the ventral aspect of the lumbosacral region.

Haematological analysis revealed leukocytosis (14.1 × 10^3 ^leukocytes/μL; normal, 4.0 to 8.8 × 10^3 ^cells/μL) and a mild increase in the activity of creatinine kinase (192 U/L; normal, 70 to 169 U/L). Cerebrospinal fluid collected via the lumbosacral space was within normal limits.

**Figure 1:**
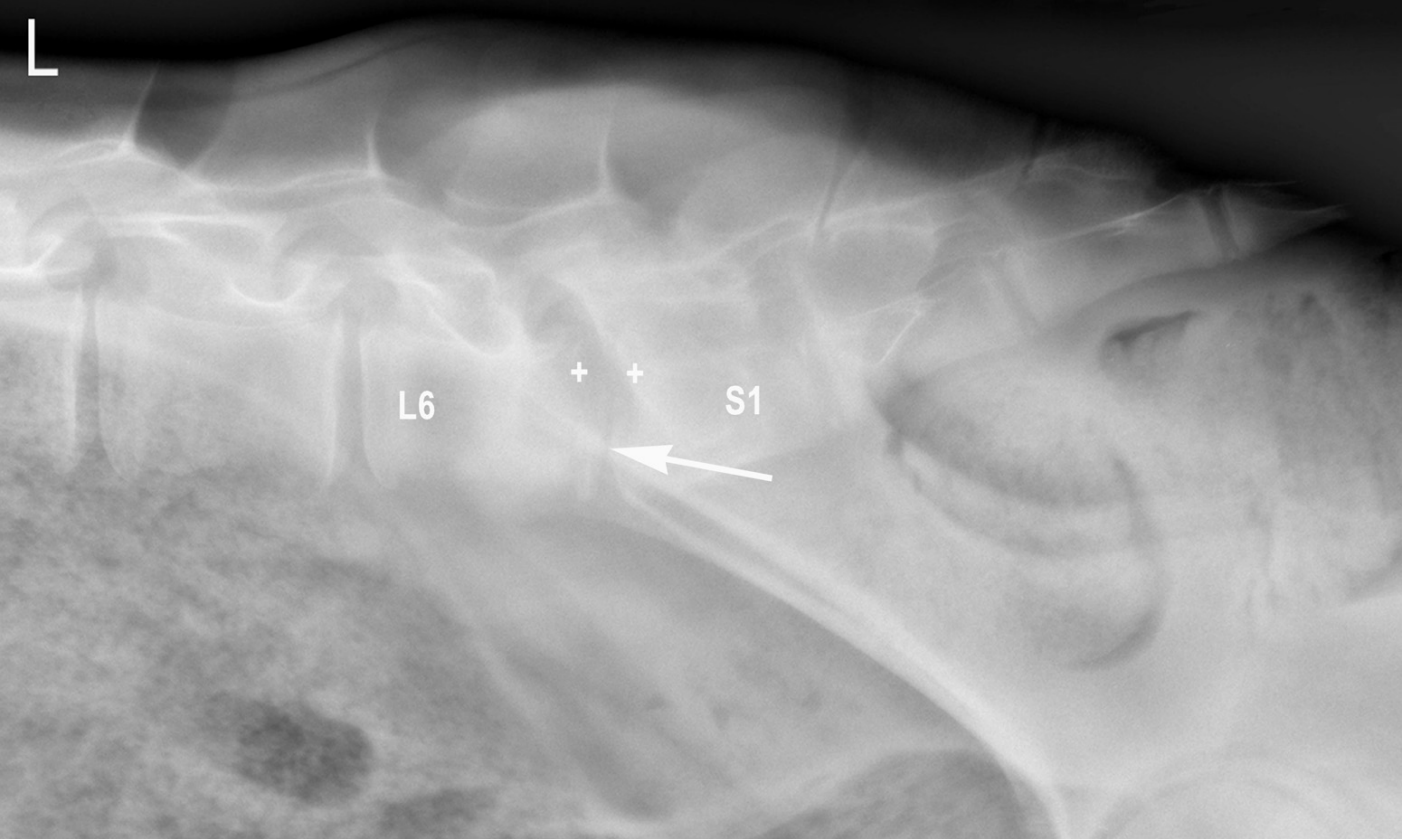
**Laterolateral radiographic view of lumbosacral discospondylitis in a 5.5-month-old heifer calf at the time of admission**. There is narrowing of the intervertebral space (arrow) and lysis predominantly of the dorsal parts of the endplates of L6 and S1 (+) and increased bone opacity adjacent to the lytic zones.

For radiographic examination, the calf was sedated with 0.2 mg/kg xylazine (Xylazin Streuli, Streuli Pharma AG, Switzerland) administered intramuscularly and anaesthetised using 2 mg/kg ketamine (Narketan^® ^10, Vétoquinol AG, Switzerland) and 0.02 mg/kg diazepam (Valium^®^, Roche Pharma AG, Switzerland) administered intravenously. The general anaesthesia allowed taking not only a laterolateral but also a ventrodorsal radiographic view. The radiographs showed narrowing of the intervertebral space between the 6^th ^lumbar (L6) and the first sacral vertebra (S1). Lysis of the dorsal parts of the endplates of L6 and S1 with sclerosis of the adjacent bone could be seen (Figure 1). According to these findings, a diagnosis of lumbosacral discospondylitis was made.

On transrectal ultrasonographic examination an irregular hyperechoic area at the ventral aspect of the junction of L6 and S1 could be detected. The hyperechoic specks were surrounded by a hypoechoic fluid accumulation and the ventral longitudinal ligament was displaced ventrally. These findings confirmed the radiological diagnosis.

### b) Treatment and outcome

Treatment was started because neurologic deficits were mild and the abnormalities of the stance could be explained by pain originating from osteolysis and soft tissue inflammation. The calf was kept in a small crate and was given 2 mg/kg cefquinom (Cobactan^® ^2.5%, Veterinaria AG, Switzerland) intramuscularly once daily for six weeks. In addition, during the first week the calf also received 3 mg/kg ketoprofen (Rifen 10%, Streuli Pharma AG, Switzerland) intravenously once daily, and during the first two weeks 4 mg/kg gentamicin (Vetagent^®^, Veterinaria AG, Switzerland) intravenously once daily.

As to be expected, clinical sings concerning the calf's gait and the kyphosis did not change significantly during the first four weeks of treatment. The calf spent most of the time lying and showed signs of pain when rising. The general condition and the calf's appetite improved slowly. The temperature fluctuated between 38.8°C and 39.4°C. After four weeks of treatment, the leukocyte count had decreased to 11.3 × 10^3^/μL.

**Figure 2:**
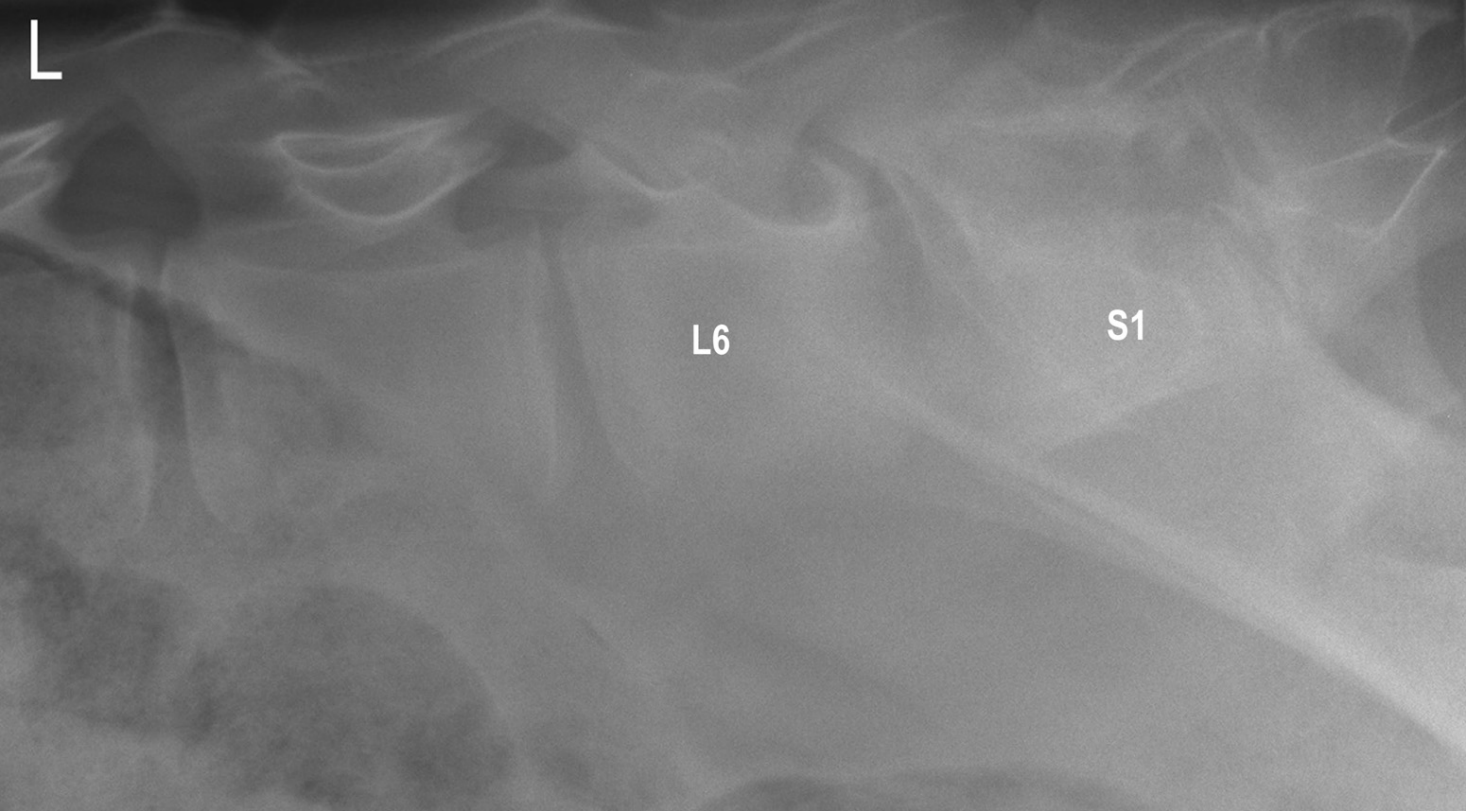
**Lateral radiographic view of lumbosacral discospondylitis in a 6-month-old heifer calf two weeks after the start of antibiotic treatment**. The endplates of L6 and S1 are completely lysed.

**Figure 3:**
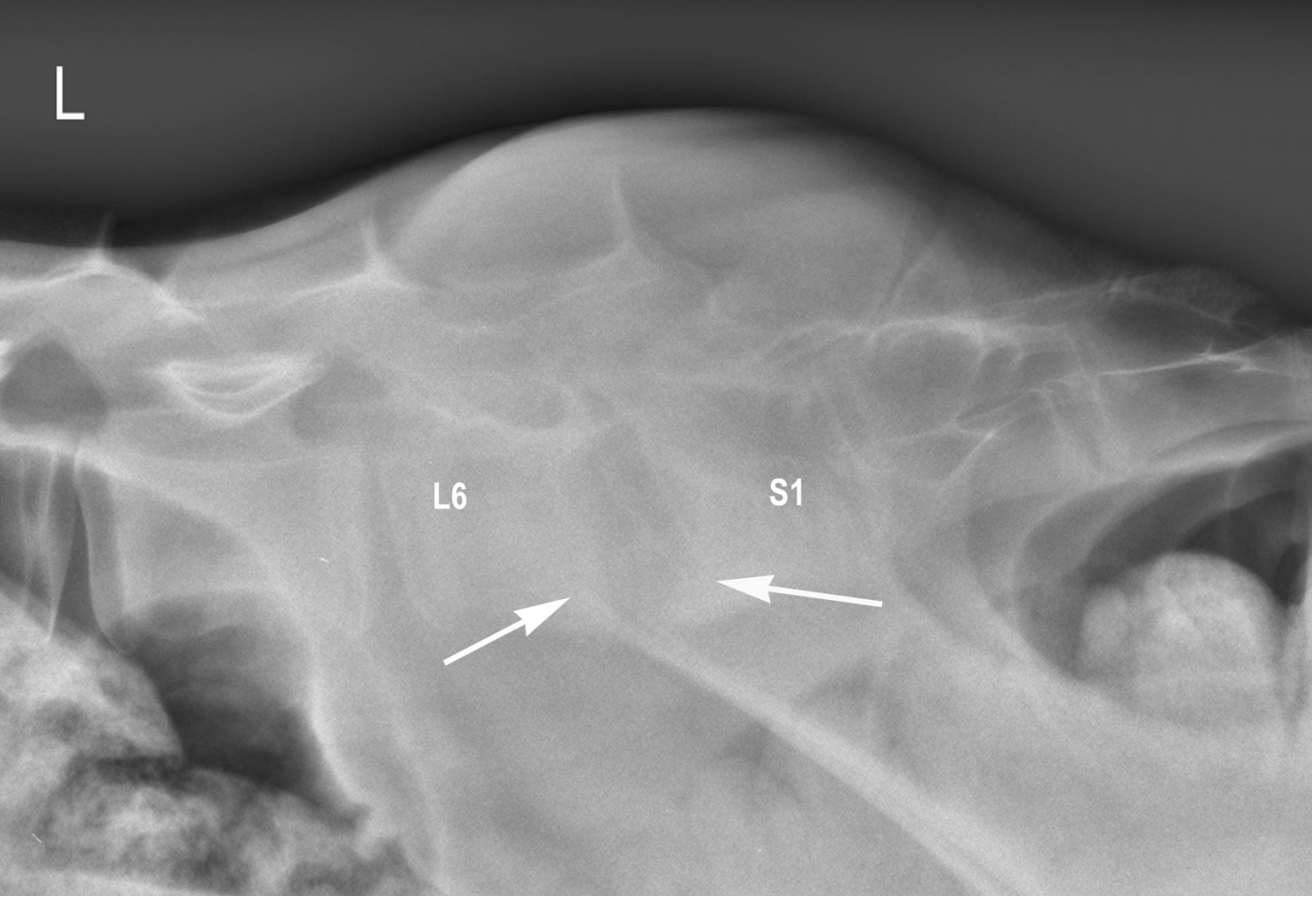
**Laterolateral radiographic view of lumbosacral discospondylitis of the same heifer calf four weeks after the start of antibiotic treatment**. In the periphery of the lytic zones, the margins of the adjacent endplate sclerosis are more defined (arrows).

Radiographs taken of the standing calf after two weeks of treatment showed increased lysis of the endplates and adjacent bodies of L6 and S1 with further widening of the intervertebral space (Figure 2). After four weeks of treatment, the size of the lytic area was unchanged and the margins of the adjacent endplate sclerosis had become more defined (Figure 3). This became even more pronounced after six weeks of treatment.

**Figure 4:**
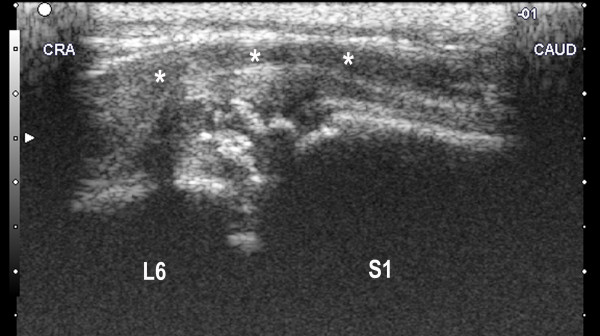
**Transrectal ultrasonogram of the lumbosacral area of the same heifer calf three weeks after the start of antibiotic treatment**. There are hyperechoic reactions ventral to the lumbosacral space surrounded by fluid accumulation in the soft tissues. The ventral longitudinal ligament is displaced ventrally (*). CRA = cranial, CAUD = caudal.

Transrectal ultrasonographic examinations were carried out every ten days. Three weeks after beginning of treatment, the hyperechoic reactions became more organised and the fluid accumulation decreased, but the ventral longitudinal ligament was still displaced ventrally (Figure 4).

Four weeks after the start of treatment, the calf began to rise on its own more frequently and with less effort, and the appetite returned to normal. Palpation of the lumbosacral area of the back still elicited pain but its severity was markedly reduced. When walking the calf for several meters in front of its crate, it moved more freely and the gait normalised. However, the calf still stood with the hind legs drawn under the body.

The calf was discharged after six weeks of treatment with the recommendation that exercise be limited to daily hand-walking for five minutes. The calf was kept in a small crate for another four months.

**Figure 5:**
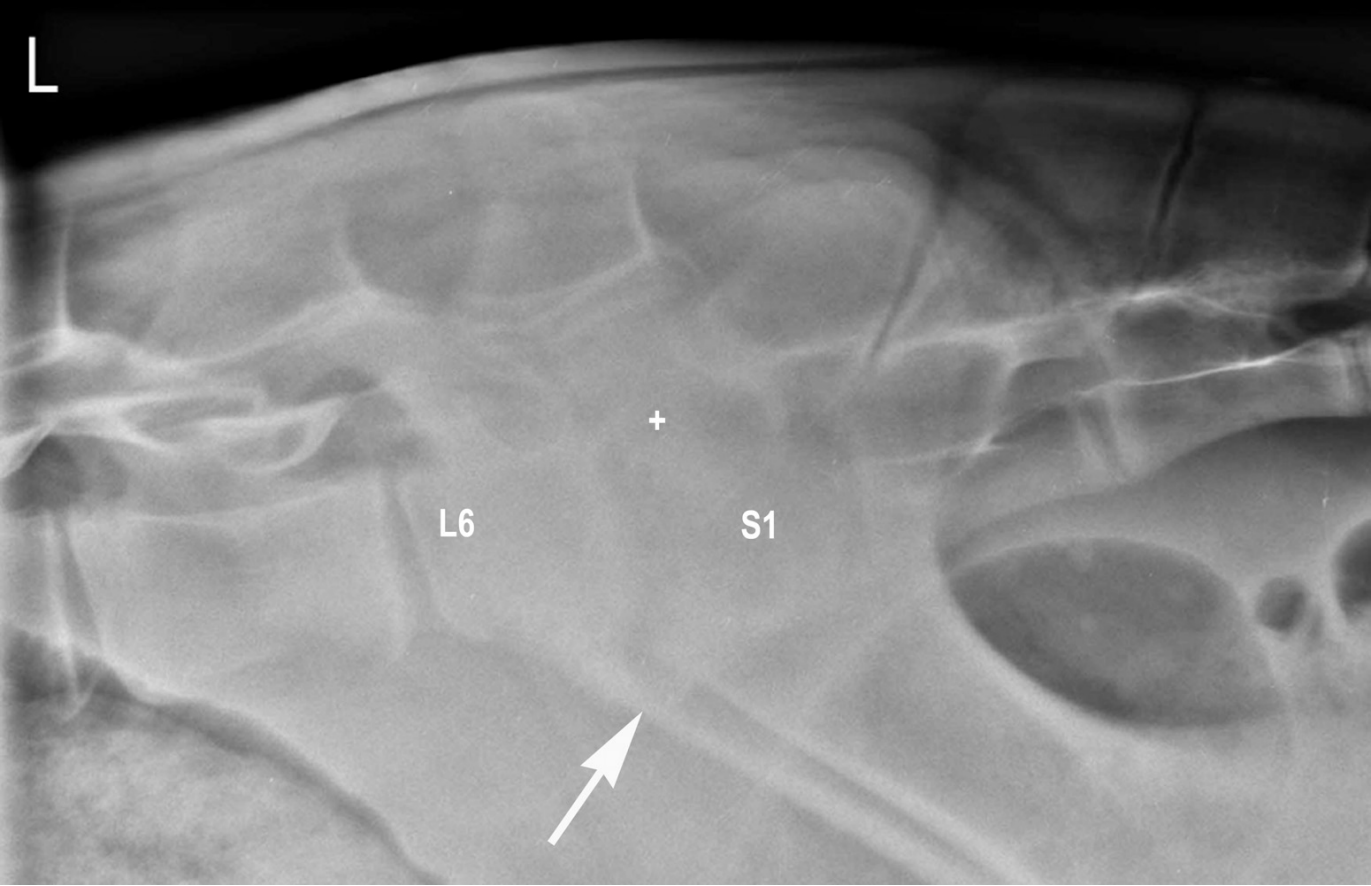
**Laterolateral radiographic view of the lumbosacral region of the vertebral column of the same heifer at 10.5 months of age, five months after initial presentation with discospondylitis**. The remodelled vertebrae L6 and S1 present with increased radioopacity and there is ventral spondylosis (arrow). The vertebrae L6 and S1 are shorter than normal and the lytic zone is much narrower than in the previous radiographs. There is a small step in the vertebral canal (+).

Clinical, radiographic and ultrasonographic examinations were repeated 3.5 months after discharge from the clinic. The general condition and demeanour were normal and the musculature of the back and hind limbs as well as the gait were the same as in other calves of the same age. The hind limbs were no longer drawn forward under the body and the palpation of the lumbosacral region of the back was not painful. During transrectal examination of the lumbosacral area, a discrete bony protuberance, 3 cm long and extending 1 cm ventrally, was felt at the junction between L6 and S1. Laterolateral radiographic views showed further remodelling of the vertebral endplates and spondylosis between L6 and S1. There was a small step in the vertebral canal (Figure 5). The spondylosis was also seen on ultrasonograms, the fluid pocket in the surrounding tissues had disappeared.

The animal was discharged and kept on alpine pasture during the summer. According to the owner, the heifer was without any problems and was pregnant 1 year after the last examination.

### c) Discussion

Discospondylitis may occur via haematogenous spread of bacteria from primary infections of the umbilicus, lungs, urinary bladder or other organs [1,4,10,12,13]. It may also occur secondary to back trauma [7] or iatrogenic infections caused by epidural or paravertebral anaesthesia [13]. However, none of these risk factors could be identified in the calf described in this report.

Percutaneous aspiration of the intervertebral space for bacterial isolation was omitted because of its potential risks. Retrospectively, it could have been performed under fluoroscopic or ultrasonographic guidance while the calf was in general anaesthesia [10]. A bacterial blood culture - provided it had been positive - would have added useful etiological and therapeutic information and possibly would have allowed a specific and potentially cheaper antimicrobial treatment. A variety of organisms including Gram-negative and Gram-positive as well as aerobic and anaerobic bacteria have been associated with discospondylitis in animals. In a study of cattle suffering from cervical discospondylitis, *Aspergillus fumigatus*, *Bacteroides nodosus*, *Clostridium perfringens*, α-haemolytic streptococci and *Staphylococcus epidermidis *have been isolated from the affected vertebrae [3]. The calf did not have a history of urogenital tract infection nor did it show any signs of an infection. Whenever there is a suspicion of urinary tract infection in an animal with discospondylitis, a bacterial urinary culture should be performed prior to antimicrobial treatment [14].

Aspirated or swallowed foreign bodies, such as grass awns, have been identified as a cause of discospondylitis in dogs. The foreign bodies migrate through the body and may reach an intervertebral space causing infection [10,15,16]. A foreign body could not be ruled out as the cause of discospondylitis in this case, but it appeared unlikely.

Different animal species appear to have different predilection sites for discospondylitis. In cattle, the location may be cervical, thoracic or lumbosacral [3-5]. In dogs, the greater mobility of the lumbosacral space compared with other intervertebral spaces has been suggested as the cause for a predilection of disease at this site [10,17].

Animals with advanced lumbosacral discospondylitis often remain in a dog-sitting position for extended periods of time during attempts to rise. The further the changes of the lumbosacral discospondylitis progress, the more neurological deficits (e.g. loss of sensitivity and proprioception, posterior paresis or paralysis, cauda equina syndrome) and muscle atrophy [3,4,13,17] may occur. In our case, differential diagnoses such as meningitis, vertebral fracture or luxation, vertebral anomaly, spinal abscess, oedema, haemorrhage or neoplasia in the vertebral canal were less likely based on clinical, radiographic and cerebrospinal fluid examinations.

Advanced diagnostic techniques such as myelography, computed tomography, magnetic resonance imaging and scintigraphy are rarely used in cattle for economic reasons, because of the large body size of the patients and because of food safety considerations in the case of bone scintigraphy. They are described in literature in different species [7,8,12,13,18] and may be very helpful in detecting multiple spinal lesions [11]. In the present case, computed tomography, although possible, was not an option because of financial restraints. Transrectal ultrasonography proved to be a useful ancillary diagnostic method, which also allowed to identify bony proliferation and involvement of adjacent soft tissues as well as to monitor healing [8]. Radiographic diagnosis was possible in the present heifer due to the small body size and the classical appearance of the radiographic findings.

In dogs, radiographic signs of discospondylitis are usually not apparent until two to three weeks after the onset of clinical signs [1]. Similar to the healing process described for dogs with discospondylitis [2,11], there was progressive osteolysis of the involved vertebrae after the start of treatment despite improvement of clinical signs in our calf. Sclerosis in the periphery of lytic lesions and bridging between the two affected vertebrae has also been seen during the healing process in cattle, dogs and horses with discospondylitis [1-3,8,11]. Complete fusion of the two affected vertebrae may occur at the end of the healing process [14].

We assumed that the calf in this report was referred to us at a relatively early stage of the disease, which may have contributed to the successful outcome. Medical treatment is recommended in affected dogs that do not have clinical or radiographic signs of instability of the vertebral column or compression of the spinal cord. If these signs occur, decompression, curettage and/or surgical stabilisation are recommended in small animals [1,10,17,19]. At this stage of the disease, the prognosis is guarded to poor in horses and cattle [3,8,18].

In dogs and horses, the administration of broad-spectrum bactericidal antibiotics for at least six weeks is considered the treatment of choice [1,2,10,17,18]. The prognosis is improved considerably with early and long-term treatment.

## Conclusions

This report shows that recovery from lumbosacral discospondylitis is possible in heifers, provided that treatment is started before major neurologic deficits have developed and is continued for an extended period of time.

### Consent

Orally informed consent was obtained from the owner of the patient for publication of this case report and any accompanying images.

## Authors' contributions

EM and BS carried out the clinical examination. TS performed the ultrasonographic examination and supervised together with KN the clinical examination and the course of the case. RH interpreted all radiographic images. EM reviewed the literature and together with KN prepared the manuscript. All authors read and approved the final manuscript.

## References

[B1] Lorenz MD Kornegay JN Oliver JE Jr, Lorenz MD, Kornegay JN Pelvic Limb Paresis, Paralysis, or Ataxia Handbook of Veterinary Neurology 2004 4 St. Louis, Missouri: Saunders Elsevier 156 158

[B2] Shamir MH Tavor N Aizenberg T Radiographic findings during recovery from discospondylitis Vet Radiol Ultrasound 2001 42 496 503 10.1111/j.1740-8261.2001.tb00976.x 11768515

[B3] Braun U Gerspach C Flückiger M Grest P Clinical and radiographic findings in six cattle with cervical diskospondylitis Vet Rec 2003 152 630 632 10.1136/vr.152.20.630 12790169

[B4] Hammond G Van Winden W Philbey A Diskospondylitis and umbilical abscessation in a calf Vet Rec 2006 158 600 601 10.1136/vr.158.17.600 16648443

[B5] Figueiredo MD Perkins GA Ospina PA Brognano C Case Report - Diskospondylitis in two first-calf heifers Bov pract 2004 38 31 35

[B6] Sura R Creden A Van Kruiningen HJ Pseudomonas-associated discospondylitis in a two-month-old llama J Vet Diagn Invest 2008 20 349 352 10.1177/104063870802000316 18460625

[B7] Zanolari P Konar M Tomek A Hoby S Meylan M Paraparesis in an adult alpaca with discospondylitis J Vet Intern Med 2006 20 1256 1260 10.1111/j.1939-1676.2006.tb00735.x 17063729

[B8] Sweers L Carstens A Imaging features of discospondylitis in two horses Vet Radiol Ultrasoun 2006 47 159 164 10.1111/j.1740-8261.2006.00123.x 16553148

[B9] Thompson K Maxie MG Bones and joints Pathology of Domestic Animals 2007 1 5 Saunders Elsevier 173

[B10] Betbeze C McLaughlin R Canine diskospondylitis: Its etiology, diagnosis, and treatment Vet Med 2002 673 681

[B11] Bahr A Thrall D The vertebrae Textbook of Veterinary Diagnostic Radiology 2007 5 St. Louis, Missouri: Saunders Elsevier 183 184

[B12] Healy AM Doherty ML Monaghan ML McAllister H Cervico-thoracic vertebral osteomyelitis in 14 calves Vet J 1997 154 227 232 10.1016/S1090-0233(97)80027-5 9414955

[B13] Zani DD Romano L Scandella M Rondena M Riccaboni P Morandi N Lombardo R Di Giancamillo M Belloli AG Pravettoni D Spinal epidural abscess in two calves Vet Surg 2008 37 801 808 10.1111/j.1532-950X.2008.00454.x 19121177

[B14] LeCouteur R Grandy J Ettinger S, Feldman E Diseases of the spinal cord Textbook of Veterinary Internal Medicine - Diseases of the Dog and Cat 2005 1 6 St. Louis: Saunders Elsevier 843 887

[B15] Johnston DE Summers BA Osteomyelitis of the lumbar vertebrae in dogs caused by grass-seed foreign bodies Aust Vet J 1971 47 289 294 10.1111/j.1751-0813.1971.tb15495.x 5106527

[B16] Gassner G Tipold A Fehr M Diagnosis, therapy, and long-term results of a foreign body-induced discospondylitis in a dachshound Tierarztl Prax 2001 29 224 228

[B17] Auger J Dupuis J Quesnel A Beauregard G Surgical treatment of lumbosacral instability caused by discospondylitis in four Dogs Vet Surg 2000 29 70 80 10.1111/j.1532-950X.2000.00070.x 10653497

[B18] Hillyer MH Innes JF Patteson MW Barr ARS Discospondylitis in an adult horse Vet Rec 1996 139 519 521 10.1136/vr.139.21.519 8953693

[B19] Adamo PF Cherubini GB Discospondylitis associated with three unreported bacteria in the dog J Small Anim Pract 2001 42 352 355 10.1111/j.1748-5827.2001.tb02473.x 11480903

